# Patients’ perceptions of targeted breast ultrasound and digital breast tomosynthesis in the diagnostic setting: A mixed methods study

**DOI:** 10.1371/journal.pone.0308840

**Published:** 2024-08-14

**Authors:** Carmen C. N. Siebers, Linda Appelman, Lejla Kočo, Mette Palm, Linda Rainey, Mireille J. M. Broeders, Peter T. M. Appelman, Shirley Go, Marja C. J. Van Oirsouw, Ritse M. Mann

**Affiliations:** 1 Department of Medical Imaging, Radboud University Medical Center, Nijmegen, The Netherlands; 2 Radboud Institute for Health Sciences, Radboud University Medical Center, Nijmegen, The Netherlands; 3 Department for Health Evidence, Radboud University Medical Center, Nijmegen, The Netherlands; 4 Dutch Expert Centre for Screening, Nijmegen, The Netherlands; 5 Department of Radiology, St. Antonius Hospital, Utrecht, The Netherlands; 6 Department of Radiology, Noordwest Ziekenhuisgroep, Alkmaar, The Netherlands; 7 Patient Advocate on Behalf of the Dutch Breast Cancer Society (Borstkanker Vereniging Nederland), Utrecht, The Netherlands; 8 Department of Radiology, The Netherlands Cancer Institute, Amsterdam, The Netherlands; Nelson Mandela African Institute of Science and Technology, UNITED REPUBLIC OF TANZANIA

## Abstract

**Background:**

Although DBT is the standard initial imaging modality for women with focal breast symptoms, the importance of ultrasound has grown rapidly in the past decades. Therefore, the Breast UltraSound Trial (BUST) focused on assessing the diagnostic value of ultrasound and digital breast tomosynthesis (DBT) for the evaluation of breast symptoms by reversing the order of breast imaging; first performing ultrasound followed by DBT. This side-study of the BUST evaluates patients’ perceptions of ultrasound and DBT in a reversed setting.

**Methods:**

After imaging, 1181/1276 BUST participants completed a survey consisting of open and closed questions regarding both exams (mean age 47.2, ±11.74). Additionally, a different subset of BUST participants (n = 29) participated in six focus group interviews 18–24 months after imaging to analyze their imaging experiences in depth.

**Results:**

A total of 55.3% of women reported reluctance to undergoing DBT, primarily due of pain, while the vast majority also find bilateral DBT reassuring (87.3%). Thematic analysis identified themes related to 1) imaging reluctance (*pain/burden*, *result*, and *breast harm*) and 2) ultrasound and DBT perceptions. Regarding the latter, the theme *comfort* underscores DBT as burdensome and painful, while ultrasound is largely perceived as non-burdensome. Ultrasound is also particularly valued for its interactive nature, as highlighted in the theme *interaction*. *Perceived effectiveness* reflects women’s interest in bilateral breast evaluation with DBT and the visibility of lesions, while they express more uncertainty about the reliability of ultrasound. *Emotional impact* portrays DBT as reassuring for many women, whereas opinions on the reassurance provided by ultrasound are more diverse. Additional themes include *costs*, *protocols* and *privacy*.

**Conclusions:**

Ultrasound is highly tolerated, and particularly valued is the interaction with the radiologist. Nearly half of women express reluctance towards DBT; nevertheless, a large portion report feeling more confident after undergoing bilateral DBT, reassuring them of the absence of abnormalities. Understanding patients’ perceptions of breast imaging examinations is of great value when optimizing diagnostic pathways.

## Introduction

A variety of breast imaging procedures are available for the detection and diagnosis of breast cancer in clinical settings. Digital mammography (DM), or Digital Breast Tomosynthesis (DBT) which replaced DM gradually from 2012 in the Netherlands, is generally considered the baseline examination [1]. DBT represents the 3D variant of conventional 2D DM, enhancing the diagnostic performance of breast cancer examinations [2]. DBT is currently the primary imaging modality for evaluating focal breast symptoms in women above a specific age, depending on national guidelines. In the Netherlands, all women over 30 years old undergo DBT as the initial evaluation, virtually always followed by targeted ultrasound (US) at the site of the breast symptom (except for pregnant or lactating women, who undergo US only) [3, 4]. Despite DBT being the standard initial modality, the importance of US has significantly increased in recent decades. Several studies have shown that US now has a better sensitivity and diagnostic accuracy than DM, and possibly also DBT, in clinical settings, along with a very high negative predictive value (NPV) (almost 100%), especially in younger women [5–10]. Recent results from the Dutch Breast UltraSound Trial (BUST) demonstrate that US is an accurate initial imaging modality in symptomatic women (not referred from screening) of all ages and outperforms DBT [11].

The BUST was designed to assess whether targeted US as stand-alone diagnostic imaging modality is also feasible for evaluating focal breast symptoms. The study showed that radiologists could rule out the presence of breast cancer with US alone in the majority of women, with a sensitivity, specificity and NPV of 98.5%, 91% and 99.8% respectively. More detailed information on the trial is provided in the original article [11]. Following the outcome of this trial, the authors suggested starting breast imaging with targeted US, performing additional DBT only in case of suspect US findings. This policy has been partially implemented in several of the participating hospitals.

Although DBT and US are thus the most common diagnostic modalities for the diagnostic work-up of women with breast symptoms in the Netherlands, little is known about patients’ experiences with these exams. Existing literature on patients’ perceptions with diagnostic breast imaging have primarily focused on DM, particularly in the screening setting. Studies examining women’s experiences with diagnostic DM have also predominantly focused on pain, revealing a wide range of pain perceptions among participants: while some patients find DM uncomfortable, others describe it as extremely painful [12]. Results across studies are also quite divergent, depending on the pain scale used. One study employed a six-options pain scale and reported relatively low pain reports (34%), while a study with a 4-point scale found some degree of pain in 66.5% of women. When using the Visual Analog Scale (VAS), the prevalence of pain ranged from 23% to 49%, depending on the use of a cut-off score of either 60 or 40 on a scale of 100 [13, 14].

The performance of DBT is similar to that of DM. However, compression may be different (slightly less, but longer). Moreover designs of modern DBT machines optimize ergonomics for patients, hence it could well be that the experiences have shifted with the introduction of DBT. Whether this is reflected in different pain perceptions in women undergoing diagnostic DBT is yet unknown. To our knowledge, only one study has investigated women’s experiences with DBT, but it focused solely on self-reported pain and was conducted among women attending screening. In this study, reported pain scores were generally low [15].

Other studies have indicated that factors influencing women’s experience with DM include fear, anticipation of results, the physical environment (e.g. waiting room), concerns about the safety and reliability of the DM procedure, satisfaction with the radiographer, and the manner in which information and results are communicated [16, 17]. Whether these results can directly be copied to the use of DBT is uncertain. Still, another study focusing on the entire diagnostic procedure, including US, highlights the significance of communication between patients and radiologists or radiographers. Therefore, clear information, personal support, and effective collaboration between patient and health care professionals, as well as among different professionals, appear to contribute to a positive experience with all modalities for patients [18].

There is also a gap in literature regarding patients’ experiences with diagnostic breast US. This is particularly relevant given the BUST results suggesting the use of US as the initial imaging modality in symptomatic women in clinical settings. Due to the absence of data of women’s perceptions regarding undergoing DBT and US, this study aims to explore these experiences within the context of the clinical work-up of breast symptoms in Dutch women who participated in the BUST trial.

## Materials and methods

### Design

The current study was conducted as a side-study to the BUST, aimed at exploring women’s perceptions of US and DBT in a reversed setting. Data were obtained using two distinct methods: 1) a questionnaire with closed-ended and open-ended questions and 2) focus group interviews. Data from the two methods were collected sequentially, analyzed separately and then integrated. This constitutes a sequential mixed-methods design study. Ethical approval was waived by the regional ethical committee CMO Arnhem-Nijmegen (2016–3034). Research data were anonymized and stored on a secure network drive in the hospital.

### Procedure

The study was conducted across three Dutch hospitals: Radboudumc Nijmegen (RUMC), Noordwest Ziekenhuisgroep Alkmaar (NWZ) and St. Antonius Hospital Utrecht (ANT). Within the BUST trial, the breast evaluation process deviated from the standard procedure. At RUMC the actual order of the procedures was reversed, with US performed prior to DBT. At NWZ, although DBT was conducted before US, the radiologist evaluated the US and DBT images in reverse order, with the DBT images only released for radiological assessment after US performance, maintaining the actual order. ANT initially adopted the NWZ model but transitioned to the RUMC model after evaluating 112 patients. US was targeted to the symptomatic area for all participants. Also, in the Netherlands, US is exclusively performed by a radiologist. Each patient provided written informed consent to participate in the BUST. Upon enrollment, all women were assigned a unique study number to anonymize their data. After imaging, the first 1276 patients enrolled in BUST were requested to complete a questionnaire regarding their perceptions of both imaging modalities.

Focus group participants were a distinct subset of BUST participants who did not complete the survey and were exclusively drawn from RUMC and ANT patients. This was because the actual reversal of US and DBT during their hospital visit, as opposed to NWZ patients for whom no changes in the imaging procedure occurred, best approximated the diagnostic pathway proposed by the BUST researchers. This patient sample was therefore best capable of reflecting on their experiences with this altered pathway. These women provided additional informed consent for participation in focus group interviews and were also asked to complete an additional questionnaire. The interviews were conducted via the online platform Zoom Meetings, during which the women were questioned about their experiences with the imaging procedure. The sessions were moderated by one of the authors (CS). Each focus group interview lasted between 90 to 120 minutes and was limited to a maximum of six women to facilitate purposeful conversation within the online environment. All interviews were recorded.

### Survey participants

The study base consisted of women who responded to the survey and were participants of the BUST trial (>30 years old) visiting the radiology departments of RUMC, NWZ, and ANT between September 2017 and September 2018 for the evaluation of focal breast symptoms. Exclusion criteria were based on the original BUST trial, which included: gene mutation carriers, men, individuals with a relevant history of breast cancer or breast surgery, or those referred from the breast cancer screening program. Women who only underwent US during the breast examination were also excluded [11]. A total of 1276 women were invited to complete a questionnaire immediately after the imaging procedure, of whom 1181 completed it.

### Focus group interview participants

Focus group participants were BUST participants who visited RUMC or ANT between September 2018 and March 2019 and had not completed the questionnaire. Participants were invited for participation approximately 18–24 months after breast imaging. Purposive sampling was performed by inviting former BUST participants who consented to be approached for follow-up studies. Inclusion criteria were patients with benign outcomes after imaging (BI-RADS 1/2 or BI-RADS 3 with negative biopsy or follow-up outcomes). This resulted in a sample of women of different age-groups (>30 years old) and different imaging outcomes (based on BI-RADS scores) to ensure a variety of perspectives were represented during the focus group discussions. Women diagnosed with breast cancer after imaging evaluation were excluded (i.e. not invited for interview participation), because US-only diagnostics would not apply to them as they would have proceeded to DBT anyway. In total, 10% of women fell into this category [11].

### Measures

Age, type of breast symptom(s) and cup size were collected for all survey participants. The survey comprised closed and open-ended questions (see S1 Appendix). In the closed questions, patients were asked about their reluctance to undergo US and DBT (yes/no). Furthermore, patients could indicate their feelings about undergoing bilateral DBT while experiencing symptoms in a single breast (comforting/unnecessary and painful/ superfluous X-radiation/neutral). In the open-ended questions section, women were asked to: 1) provide an explanation for their reluctance to US and DBT if they had reported this, and 2) describe their overall experiences with US and DBT.

For focus groupparticipants, information on their age, marital status, ethnicity, education level, past and (intended) future participation in breast cancer screening, and whether they have experiences with breast cancer in their personal environment was collected. The focus group discussions mainly focused on women’s experiences with DBT and US (see S2 Appendix).

### Statistical analysis

Descriptive statistics for women’s responses to the closed-ended survey questions are presented in the Closed Questions section of the Results. Descriptive analyses were conducted using IBM SPSS Statistics Version 25.

To organize the responses from the open-ended survey data and focus group interviews, thematic analysis was used [19]. This is a widely used method to identify, analyze and interpret patterns within a dataset. Braun and Clarke argue that thematic analysis can be viewed as a foundational method for performing qualitative analysis, rather than a methodology with a singular theoretical and epistemological basis. It enables researchers to succinctly and flexibly summarize key features in all types of data in a structured manner.

In addition to thematic analysis to explore patterns of meaning within the data, content analysis was utilized to convert the qualitative survey data into quantitative form by tallying the number of coded lines within each (sub)theme in the questionnaire [19]. Both the absolute number of times the topic was mentioned and the percentages were provided. A maximum of 2362 coded lines within each (sub)theme was possible (2 x n = 1181 for both US and DBT). Percentages of the number of times a topic was mentioned were based on this total number of lines.

Because the interviews were very loosely structured, we refrained from quantifying of this data. Instead, we aimed to determine whether women’s experiences as expressed in the interviews corresponded with the survey data. This could be seen as a form of cross-checking data. Interview quotes were thus complementary to the survey data and not tallied or included in the percentages.

The steps in thematic analysis include familiarizing with the data, coding the answers, creating themes and reviewing these themes, defining and naming the themes, and conducting the final analysis to create reliable categories [19]. Two researchers independently went through these steps for both the questionnaire (CS and LK) and interview data (CS and MP). Consensus was subsequently reached through discussion or after consultation with a third researcher (RM), who acted as a final assessor when discrepancies arose.

## Results

A total of 1181 patients completed the questionnaire, resulting in a participation rate of 92.6%. Their ages ranged from 30 to 90 years old, with a mean age of 47.2 years (SD = 11.74). The majority of patients presented with a palpable lump (74.3%). Among all patients, 181 underwent biopsy of a lesion that was causing the breast symptom (15.3%). Characteristics of all patients that completed the questionnaire are presented in Table 1 (full patient data available in S3 Appendix).

**Table 1:** Survey participant characteristics.

	N	*(%)*		N	*(%)*
**Age categories**			**Biopsy of the breast symptom**		
30–39	332	*(28*.*1)*	No	1000	*(84*.*7)*
40–49	464	*(39*.*3)*	Yes	181	*(15*.*3)*
50–75	353	*(29*.*9)*	**Cup size**		
>75	32	*(2*.*7)*	AA	3	*(<1*.*0)*
**Hospital**			A	110	*(9*.*3)*
Radboud UMC	246	*(20*.*8)*	B	304	*(25*.*7)*
NWZ	446	*(37*.*8)*	C	284	*(24*.*0)*
Antonius Hospital	489	*(41*.*4)*	D	238	*(20*.*2)*
**Breast symptom**			E	121	*(10*.*2)*
Lump	877	*(74*.*3)*	F	45	*(3*.*8)*
Focal pain	394	*(33*.*4)*	G	20	*(1*.*7)*
Focal itch	12	*(1*.*0)*	Missing	56	
Nipple retraction	33	*(2*.*8)*			
Nipple discharge	23	*(1*.*9)*			
Other	63	*(5*.*3)*			
Missing	15				

A total of 29 women participated in six interviews, with 3–6 women per focus group. These interviews took place between October and December 2020. The patients’ ages ranged from 33 to 63 years old, with a mean age of 48.4 years (SD = 8.3). Additional information on their characteristics can be found in Table 2 (full patient data available in S4 Appendix).

**Table 2:** Focus group interview participant characteristics.

	N	*(%)*		N	*(%)*
**Age category**			**Ethnicity**		
30–39	4	*(13*.*8)*	Dutch	23	*(79*.*3)*
40–49	12	*(41*.*4)*	Turkish	1	*(3*.*4)*
50–75	13	*(44*.*8)*	Western European	1	*(3*.*4)*
**Hospital**			Spanish	1	*(3*.*4)*
RUMC	16	*(55*.*2)*	Moroccan	1	*(3*.*4)*
ANT	13	*(44*.*8)*	Italian	1	*(3*.*4)*
**Marital status**			Missing	1	
Married	19	*(65*.*5)*	**Past screening participation**		
Single	4	*(13*.*8)*	Yes	16	*(55*.*2%)*
Living together	3	*(10*.*3)*	No	12	*(41*.*4)*
Divorced	1	*(3*.*4)*	Missing	1	
Widowed	1	*(3*.*4)*	**Future screening participation**		
Missing	1		No	25	*(86*.*2)*
**Education**			Yes	3	*(10*.*3)*
Secondary	3	*(10*.*3)*	Missing	1	
Vocational	5	*(17*.*2)*	**Breast cancer in personal environment**		
Higher vocational	11	*(37*.*9)*	Yes	24	*(82*.*8)*
University	7	*(24*.*1)*	No	4	*(13*.*8)*
Missing	3		Missing	1	

### Closed survey questions

#### Reluctance to US and DBT

In total, 8.3% of patients reported being reluctant to undergoing US (response rate 97.2%). Regarding DBT, 53.6% expressed reluctance toward the examination (response rate 96.9%). Reasons for reluctance to the examinations are described in the next section.

#### Usefulness of bilateral DBT

There was a response rate of 94.8% on the survey item on usefulness of bilateral DBT. Despite common reluctance, most patients believe that bilateral DBT is reassuring (82.7%). Only a fraction of women are of the opinion that it is either an unnecessary and painful examination or that it leads to superfluous X-radiation (respectively 1.9% and 1.7%). Additionally, 8.6% of women expressed a neutral opinion. Data of the closed questions are available in S3 Appendix.

#### Open survey questions and focus group interview results

**Fig 1:**
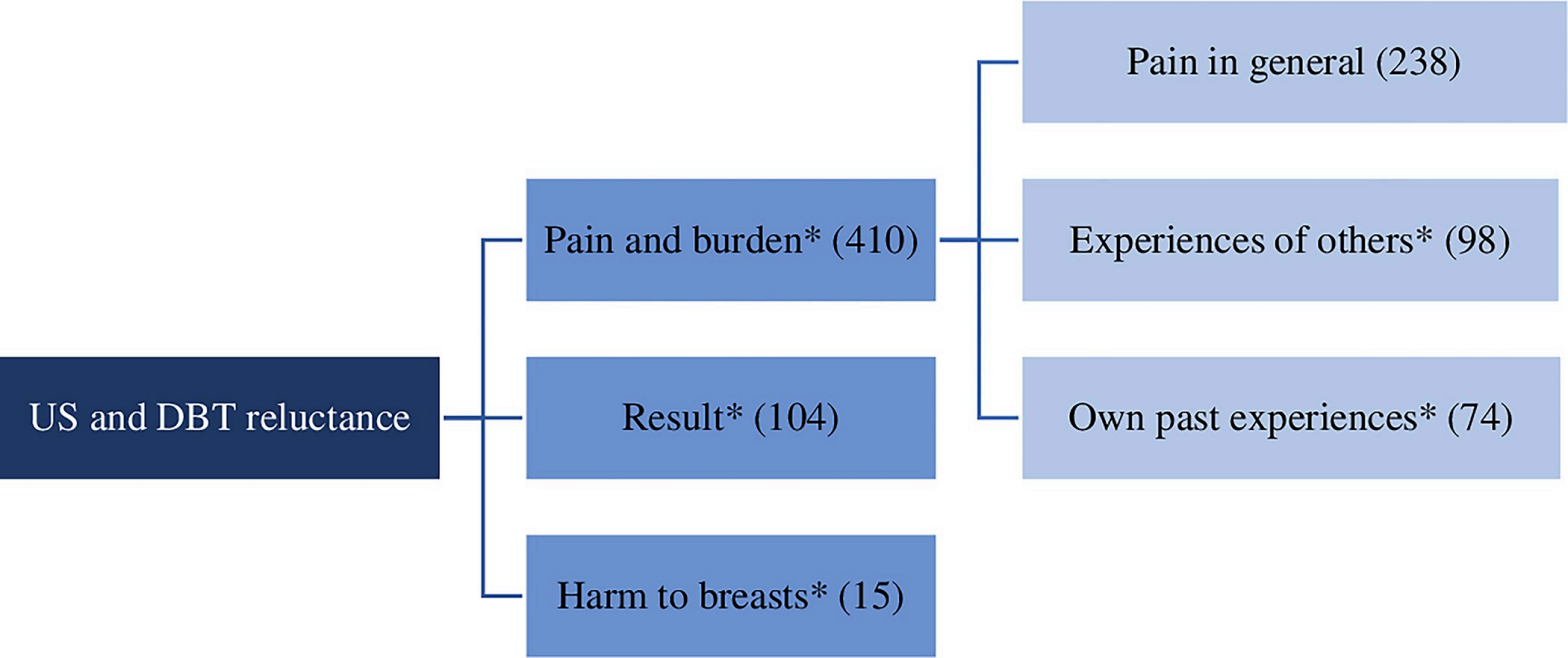
Patients’ reason for US or DBT reluctance with number of coded lines from survey data within each (sub)theme between parenthesis. * Theme emerged from both open-ended survey questions and interview data.

**Fig 2:**
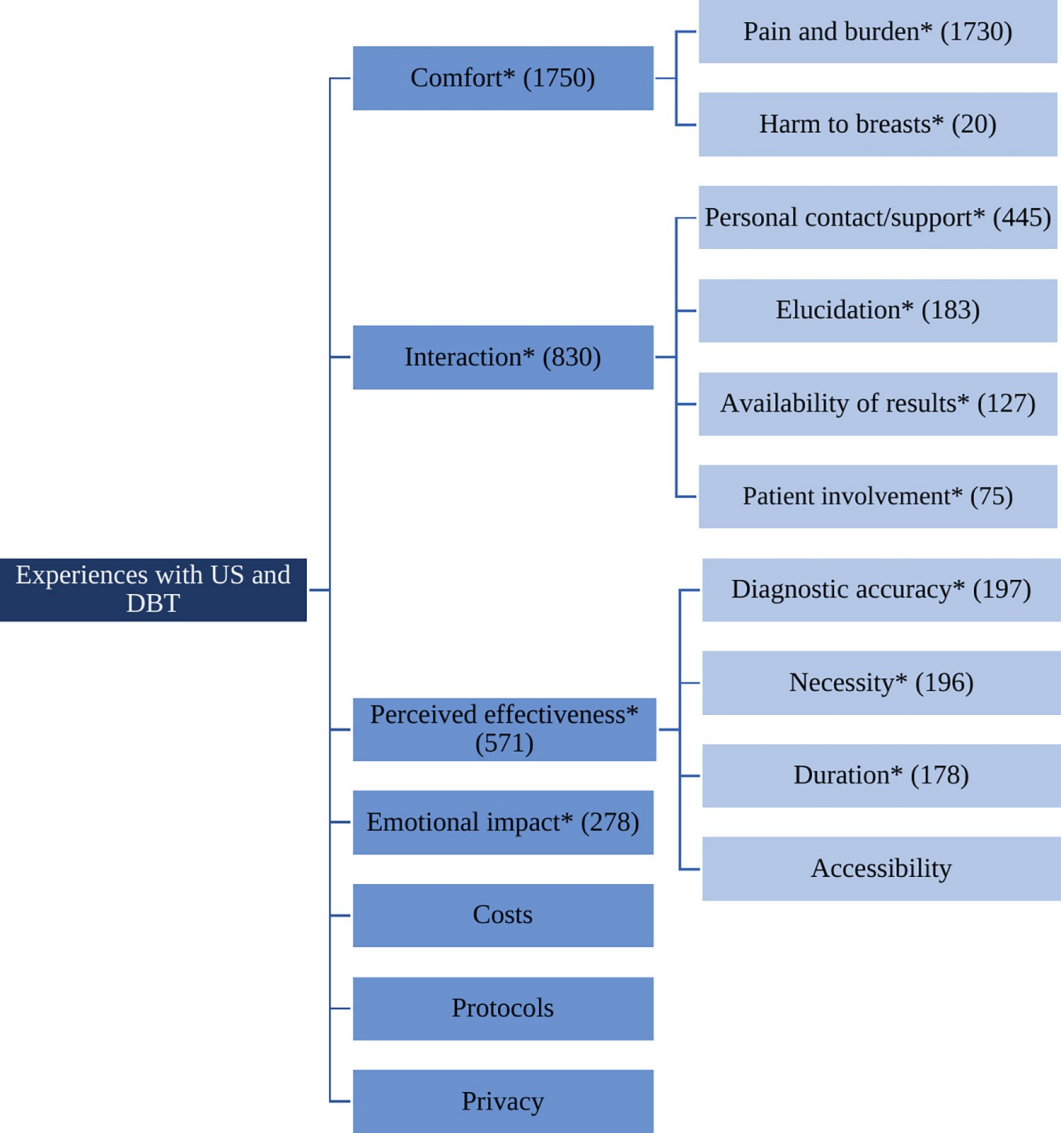
Patients’ experiences with US and DBT with number of coded lines within each (sub)theme between parenthesis. * Theme emerged from both open-ended survey questions and interview data.

Two groups of themes emerged from the data: 1) themes on reluctance prior to the exams, and 2) themes on actual experiences with the exams. Themes related to US or DBT reluctance as identified from the survey data, included pain and burden of the exam, the result, and the potential harm to their breasts. The main themes regarding women’s experiences with both modalities included comfort, interaction, perceived effectiveness, emotional impact, privacy, costs and protocols. The themes and subthemes are described in Figs 1 and 2 (* denotes themes that emerged from both the survey and interview data).

#### Themes related to reluctance to undergo imaging

*Theme 1*. *Pain or burden*. For US, the number of patients anxious about potential pain or burden is very small; only four women (<1%) reported being “Afraid of pain” or feeling “Ill at ease”. In contrast, fear of DBT is much more common due to pain (n = 406, 17.2%). Some patients were unclear about the source of their fear, but simply reported being reluctant to undergo DBT “because of the pain” (n = 234, 9.9%). Additionally, 98 patients (4.1%) expressed fear due to hearing about painful experiences from other women. This sentiment was echoed in the interviews, with women stating that they “know the stories […] so you hear them a lot like… it really hurts…”. Lastly, there were patients who had undergone DM or DBT before, such as at the national screening program, and they were frightened due to previous painful experiences (n = 74, 3.1%). This was also mentioned by women in the interviews: “I have had this exam two times and the second time I did not look forward to doing that mammography again”.

*Theme 2*. *Result*. Reasons for US reluctance were mostly related to stress and fear of a bad outcome, rather than reluctance towards the examination itself. Of all women, 62 (2.6%) reported to be “anxious about what would come out. I did not mind the examination itself”. Participants in the focus group interviews shared similar perceptions: “I was really anxious. I think every woman who undergoes such an exam goes there with fear that there’s something… well, that bad news will come out”. Regarding DBT, 42 women (1.8%) were reluctant due to anxiety about the result.

*Theme 3*. *Harm to breasts*. While women reported no fear of harm to the breast due to US, 15 (<1%) expressed concerns about the potential harm that DBT could cause to their breasts and health. The nature of their concern varied, from fear of harm to their breast implants (“Implants, afraid of damage”) to concerns about ionizing radiation. This latter concern was also expressed by focus group participants: “I’ve heard once that too much X-radiation, pictures, can actually cause cancer”. Other women were against the compression of the breasts between the plates due to the potential harm this could cause: “The crushing of the breast tissue doesn’t seem good to me”.

#### Themes related to experiences with both imaging methods

*Theme 1*. *Comfort*. The first theme, ‘comfort’, reflects the (dis)comfort experienced by women during US and DBT, either due to (the absence of) pain or burden, or potential harm. The first subtheme highlights the considerable number of times it was emphasized that US is not painful or burdensome (n = 324, 13.7%), and women even indicated finding it pleasant or relaxing (n = 210, 8.9%). This was confirmed numerous times in the focus groups. Another 322 neutral remarks on US were made (13.6%). Only eleven women (<1%) indicated experiencing some burden or pain due to US; as one woman in the interviews explained: “At that moment during ultrasound I didn’t find it pleasant of course, because I just don’t like it when someone touches my body”. DBT, on the other hand, was much more associated with burden or pain, as mentioned 512 times (21.7%). To illustrate, an interview participant said that “In a sense, I would rather undergo childbirth three more times than to have another mammography”. However, there was a number of women who thought the DBT exam was “not as unpleasant as expected” (n = 339, 14.4%), which was also emphasized multiple times in the focus group interviews. Only twelve women (<1%) reported a positive or pleasant experience with DBT.

Regarding the second subtheme, harm to breasts, US was perceived as hardly invasive (n = 3, <1%), while DBT was believed to be harmful to the breasts by a small number of women (n = 17, <1%). Opinions on harm were more divided in the focus group discussions, with most women expressing less fear regarding factors such as radiation: “If you get on a plane, I understand that the radiation exposure is much greater than that from a single mammogram”.

*Theme 2*. *Interaction*. The second overarching theme highlights women’s appreciation for the interactive aspect of breast imaging. This encompassed personal guidance from medical staff, explanations provided by radiologist, availability of results, and patient involvement in the examination. The first subtheme, personal guidance, was cited 445 times (18.8%) as a significant factor in both the US and DBT. As one interview participant explained, she preferred undergoing US first: “You have much more direct contact, of course, compared to being placed in such a device [DBT machine]”. It was also noted that the attitude and expertise of the radiographer contributed to the tolerability of DBT: “That’s what I experienced the first time […] just pushed between and flattened […] But the second time she was very sweet, gently compressing a bit more each time and asking, ‘can it go a bit further, can it go a bit further?’ So, I thought, in this way I dare to do it”.

As reflected in subtheme two, clear explanations by the radiologist during US were deemed important by many women (n = 183, 7.7%), with some stating, for example: “Clear explanation in advance and during the ultrasound regarding the procedure and findings is important”. This sentiment was also echoed in the focus groups: “They could tell me all kind of things about what they found and what is considered normal, and what that looks like…”.

The third subtheme reflects how readily information or results are available after imaging. While women immediately received their preliminary diagnosis during US, DBT required women to wait longer before being informed about the results (n = 127, 5.4%): “Pleasant that you first undergo the examination where the doctor can provide a first indication of whether the result is good/bad”. However, some women in the interviews emphasized that the results were also quickly available after DBT: “Well, not “right away-right away”, but maybe I had to wait for about 15 minutes or so. But it’s not as if you didn’t hear it until three days later, so to speak”.

The last subtheme illustrates the extent to which patients were involved in the exam; unlike DBT, patients are able to watch along with the imaging during US as mentioned 75 times (3.2%): “Pleasant to be able to watch and listen along with what the doctor is seeing”. One woman in the interviews also mentioned that undergoing an US raises awareness of one’s own body: “It helps in getting to know [your breasts] better, because you can feel some things, but what exactly are they?”.

*Theme 3*. *Perceived effectiveness*. The third theme that received much attention was associated with the perceived effectiveness of both modalities, with subthemes including diagnostic accuracy, necessity, duration and accessibility of the exams. According to the survey data, some women expressed more confidence in the diagnostic accuracy of US (n = 88, 3.7%) while others favored DBT (n = 25, 1%). This contrast was also evident in the focus groups. On one hand, some women mentioned, for example: “I can imagine that you cannot see very small things with ultrasound, that you can see with for example a mammogram”. On the other hand, other women had experiences with lesions that were occult on DBT: “They always say to me that not everything is seen on the mammogram, because I have difficult tissue”. Moreover, both in the survey (n = 84, 3.6%) and in the interviews, women expressed doubts about the reliability of the targeted focus of US on the symptomatic area, emphasizing the strength of bilateral DBT for screening the entire breasts: “Only the spot was looked at [with US] and not the entire breast(s). I also wanted assurance that both breasts were OK”.

The second subtheme illustrates patients’ disagreement on the necessity of both modalities for diagnostics. While some women (n = 22, <1%) believe US only would suffice, it was stated 118 times (5%) that DBT is essential and US alone is not sufficient. They say, for example: “[DBT is] Necessary to obtain a proper diagnosis. That outweighs the investigation”. Some women were more neutral about which exams were necessary (n = 43, 1.8%): “What has to be done must be done”. In 13 responses (<1%) it was explicitly suggested that the combination of US and DBT would be most adequate.

The third subtheme ‘duration’ particularly highlighted the brief duration of the US (n = 119, 5%), although this aspect was scarcely mentioned in the interviews. Women stated, for example: “Everything happens nice and fast, including biopsy”. It was noted 55 times (2.3%) that DBT proceeded “faster than expected”.

Subtheme four only emerged from the interview data and describes how initial US is more accessible and could lower the threshold for women to undergo diagnostic breast imaging: “It’s maybe cheaper, it’s more accessible, people can be reluctant to undergo mammography, because, yes, it is not a pleasant exam… You could maybe lower the threshold as a result of which maybe more people are going to say ‘well, yes, I’ll just do an ultrasound then’”.

*Theme 4*. *Emotional impact*. This theme describes the emotional impact of US and DBT. Some women who completed the survey (n = 11, <1%) as well as focus group participants agreed that the diagnostic period can be very stressful: “I was very nervous, because, of course, you’re not undergoing such an exam for no reason”. Additionally, women felt reassured by each modality to various extents. While it was mentioned frequently that US (n = 79, 3.3%) or DBT (n = 87, 3.7%) felt reassuring, in some women, both modalities evoked opposite feelings (n = 11 and n = 10 respectively, <1%). This same contrast was very prominent in the interviews. Some women said that “[US] would have comforted me directly, so I actually didn’t want to go for mammography anymore” while others preferred additional DBT: “Eventually, I found it pleasant that I got both ultrasound and mammography, because, for me, it’s like, OK everything that could have been done, was done”. Regarding the order of imaging, women said that it was reassuring to first undergo US (n = 80, 3.4%): “Because the ultrasound is done prior to the mammography, the first uncertainty is already taken away, therefore you also enter the mammography with a different feeling”. This sentiment was confirmed by many focus group participants, who noted, for example, “You are going through that exam with another feeling than when you first encounter that cold, well, sterile device with no one around”.

*Theme 5*. *Costs*. This theme highlights the potential for cost savings, both on an individual and societal level, when only performing US, as revealed in the interviews. Indeed, women expressed the belief that DBT could serve as a financial barrier to undergoing diagnostic imaging: “Of course, when you have to cover the costs of the exam yourself, I can understand that it could also pose a barrier for less fortunate people”.

*Theme 6*. *Protocols*. Theme six, which emerged solely from interview data, revolves around women’s perceptions of the imaging protocols utilized. Some women expressed the belief that the guidelines are quite rigid, while others recounted experiences where there were deviations from the original protocol. One woman articulated this by saying: “It is protocol that that mammography must be done first. This time, I mentioned that I had undergone it last week, [so] I said ‘or you just do an ultrasound?’ ‘No that is not allowed, it is protocol’”. Additionally, women were cognizant that the US evaluation was targeted to the area of concern, with a whole-breast evaluation only occurring in exceptional circumstances: “I found it odd that only one… I asked, don’t you have to do the other [breast]…?’ ‘No that’s not necessary because you only have complaints on the right side’”.

*Theme 7*. *Privacy*. The last theme encompasses all statements regarding privacy during the diagnostic procedure, as reported solely in the interviews. Some women mentioned feeling that their privacy was violated when the exam results were disclosed in the waiting room instead of in a separate area. One participant expressed her concern: “There was a woman, a somewhat older woman with her daughter who was crying in the hallway, and well, the doctor came outside and he said things that made me think… you cannot do that. I mean, where is the privacy here?”. Additionally, another woman emphasized that overhearing other women’s emotions or conversations with the doctor could also affect their own feelings towards the exams: “If I’m sitting in the waiting room and I hear that woman in the hallway has it [cancer], I still have to go. So for me it is… That step to go inside is even harder”.

## Discussion

The present study offers insight into Dutch women’s perceptions of DBT and US for assessing focal breast symptoms. It highlights both positive and negative aspects of both examinations, with notable criticism regarding strict adherence to guidelines and privacy concerns. Human interaction during exams was widely recognized as important. US was primarily associated with comfort, high patient involvement, rapid clarification of symptoms, short duration, and lower costs. DBT was perceived as more burdensome or painful, leading to reluctance, longer waiting times, and less patient involvement. Perceptions varied regarding the effectiveness and emotional impact (reassurance) of both modalities. While many women believed that US was sufficient for diagnosis, bilateral DBT was reassuring for most.

We observed that more than half of the women expressed reluctance toward DBT. Apart from the typical anxiety related to a negative outcome, this reluctance predominantly stemmed from anticipated pain. Such expectations were often based on either personal past experiences or anecdotes from other women. It is known that in women attending for screening DM a second or subsequent time, previous DM pain experiences serve as a reliable predictor for current DM discomfort [20–23]. However, for first-time attendees, the accuracy of expectations tends to be lower. Poulos and Rickard [24] show that in only 35.7% of the patients the expectation aligned with their actual experiences. Additionally, women tend to anticipate higher levels of pain when their expectations are influenced by exaggerated accounts from others, even though the reality often proves to be less severe [17]. This is also known as the nocebo effect, which indicates that “expectation of a negative outcome may lead to the worsening of a symptom” [25]. This is in line with our observations regarding diagnostic DBT. Therefore, offering accurate information by professionals, particularly to first time attendees, appears crucial in mitigating discomfort by preventing women from unrealistically high pain expectations [26].

While US is typically described as completely painless or minimally painful for nearly all women, as also shown by Prosch et al. [27], unpleasant DBT experiences remain prevalent in diagnostic imaging. A significant proportion of women in our study highlighted DBT-related pain and discomfort, although there were also women who thought the exam was not that bad. This parallels literature focusing on diagnostic DM showing it to be frequently burdensome and painful, although the intensity of pain varies within and across studies [13, 14]. DM or DBT-related discomfort is especially important in our study population, as symptomatic women might experience greater anxiety and pre-existing pain than women in the screening population. Therefore, their DBT experience might be even worse, as it is known that these factors are associated with an increased pain intensity [16, 28, 29]. Especially for this population, more attention and care from the radiographer during DM and DBT is crucial.

During both US and DBT, the aspect of interaction appears to be highly important. Indeed, listening skills, explaining the procedure, and the competence of the radiographer are shown to be essential. In the current study, the majority of women praised the radiographers’ approach, skills, and instructions. This differs from previous qualitative research, where unpleasant staff interactions were much more common [16, 17, 30, 31]. This may again result from differences in patient populations. In contrast to screening DM, where radiographers are strictly bound to time limits, more time is available for interaction in the diagnostic setting [32]. This might result in greater satisfaction with the radiographer’s approach [18]. Regarding US, women especially value the direct personal contact and explanation by the radiologist. As suggested by Mussetto et al. [33], this might provide more reassurance to the patient. Importantly, US examinations in the Netherlands are always performed by a radiologist, whereas in other countries this might also be done by qualified radiographers.

The availability of imaging results and patient involvement were frequently recurring subthemes within the interaction theme. Direct disclosure of preliminary diagnoses and immediate exclusion of breast cancer during US were found reassuring, especially as women could observe the imaging process in real-time on screen. This aligns with previous research, which demonstrates that patients’ anxiety decreases directly after US performance [34, 35]. Disclosure of DBT findings is more time-consuming, with patients unable to visualize the breast images, potentially prolonging their distress and uncertainty. Indeed, Flory and Lang [36] show that uncertainty of outcome and awaiting diagnosis has substantial impact on women’s anxiety and perceived stress levels, often leading to depressive symptoms and impairments in daily life.

Patients’ perceptions of the effectiveness of US and DBT as diagnostic instrument are diverse. While the short duration of US, in contrast to DBT was generally welcomed, apprehensions arose concerning the unilateral focus of targeted US and its diagnostic accuracy beyond the symptomatic area. A subset of women presumably seeks comprehensive assurance of the absence of abnormalities in both breasts, rather than mere elucidation of symptoms in the affected breast. Consequently, division on the necessity of both exams was observed; some women deemed DBT redundant, while others deemed it indispensable. This dichotomy likely contributes to the variance observed in the emotional impact of the imaging procedure. Over 87% of the women expressed a sense of reassurance with bilateral DBT. Concurrently, many women found solace solely through US, albeit the number of women reporting such sentiments remains modest. In addition, in the screening setting, findings suggest that while some women acknowledge the fallibility of DBT, others firmly endorse its efficacy in early breast cancer detection [30]. It is indeed acknowledged that patients often lack awareness regarding the specifics and performance metrics of their diagnostic tests, underscoring the importance of providing comprehensive information [12, 37].

Another significant aspect influencing the perceived effectiveness of the breast evaluation, as mentioned in the interviews, is related to the accessibility of diagnostic care, albeit this was only a theme within the focus group discussions. US is perceived as a less invasive exam compared to DBT, potentially reducing the barrier for women to visit the GP or hospital. This is also intertwined with considerations of lower costs when patients bear the financial burden of examinations out-of-pocket. Although no earlier studies show the impact of costs on women’s decisions to pursue *diagnostic* breast imaging, numerous studies underscore the role of financial barriers in impeding women from undergoing *screening* mammograms, particularly in regions where healthcare services are out-of-pocket expenses [38–40]. Consequently, the influence of elevated medical expenses on adherence to health care services among low-income patients should not be underestimated. Although financial barriers appear to be a minor concern in our study population, where diagnostic imaging procedures are largely financially covered (with diagnostic exams being deductible in the Netherlands [41]), these findings may hold broader international implications. This is especially relevant for underserved population groups who could benefit from the reduced costs associated with US-only evaluations.

Patients exhibited varied perceptions regarding the strict adherence to protocols at the radiology department. Most women believed that deviating from the guidelines because of patients’ preferences or reluctance is rarely done. However, there is a growing recognition of patient-centered care within both health care in general and radiology practice specifically. This means that “providers respect patients’ values and preferences, address their emotional and social needs, and involve them and their families in decision making” [42]. Within the breast clinic, radiologists could initiate this approach by prioritizing patients’ emotional concerns and providing comprehensive information regarding imaging procedures, including the rationale behind the selection of specific modalities. Moreover, patient-centered care extends to attention to the physical environment of hospitals, ensuring privacy before, during and *after* examinations [42–44].

There are several limitations to the current study and its methodologies. Firstly, we utilized a combination of closed-ended and open-ended questions, thereby integrating quantitative and qualitative data. A recognized risk of this approach is the potential for ‘priming’ effects, wherein preceding questions may influence patients’ attitudes or opinions. Responding to closed-ended questions before open-ended ones could lead to information stored in the former affecting responses given to the latter [45]. Consequently, the survey data may not fully represent patients’ genuine perspectives on the matter. Moreover, the questionnaire was conducted directly after imaging, so patients’ emotional and mental states might have affected their perception, as they had just undergone the examination.

However, these limitations were partially addressed by conducting the focus group interviews later on, involving a different cohort of BUST participants. This approach allowed women to describe their experiences with the past exam more neutrally. As the interview findings largely complemented the survey results, they strengthen our conclusions regarding women’s perceptions on the imaging tests. However, it should be noted that this study was conducted within the context of the BUST, where most women underwent US prior to DBT. Therefore, women’s experiences with these modalities in the ‘standard’ clinical setting (i.e. first DBT followed by targeted US) cannot be fully assessed.

## Conclusions

Our study contributes to existing literature by providing evidence that both US and DBT exhibit distinct advantages and drawbacks. Additionally, it offers deeper insights into patients’ perceptions of these modalities within the context of assessing focal breast symptoms. Our findings suggest that US is highly tolerated, with many women valuing its use as a first-line modality, particularly due to the direct interaction with the radiologist and the immediate explanation of the imaging findings. On the other hand, DBT is met with some apprehension due to potential discomfort, yet it offers additional reassurance to a subset of women who may not be entirely satisfied with the targeted approach of US. Adapting imaging protocols to patient preferences and expectations appears crucial for optimizing accessibility and satisfaction with imaging studies for focal breast symptoms.

## Supporting information

S1 Appendix Survey translated to English. (DOCX)

S2 Appendix Interview prompt questions. (DOCX)

S3 Appendix Closed ended survey data. (XLSX)

S4 Appendix Focus group data on patient characteristics. (XLSX)
