# Supplementary material for: Patients’ perceptions of targeted breast ultrasound and digital breast tomosynthesis in the diagnostic setting: A mixed methods study
Source: PLoS One. 2024 Aug 14;19(8):e0308840. doi: 10.1371/journal.pone.0308840 (PMC11324127; doi:10.1371/journal.pone.0308840)
Supplement: S1 Appendix — (DOCX) [file pone.0308840.s001.docx]

Study number: .........................................................................................

Date of imaging: .........................................................................................

Dear Madam,

You have been referred to the Radiology department because of breast complaints, such as a lump in the breast. In addition, you have given consent to participate in a study regarding the evaluation of medical imaging in women with breast complaints. By now, you have undergone ultrasound and mammography, besides any potential other investigations.

We would like you to ask to answer the questions below. Your opinion is important to us and will be included in the analysis of the study.

**GENERAL:**

- Indicate what applies to you.
- Breast complaints:
  - Lump
  - Nipple discharge
  - Nipple retraction
  - Focal skin retraction
  - Focal pain/odd feeling (focal = not more than a quarter of the breast)
  - Other, being…….....………………………………………………………………………………….………………..
- What is your cup size? ………………………………………………………..
- Do you have the intention (in case you have not reached the age of 50 yet) or do you participate in the national breast cancer screening program? YES/ NO

**ULTRASOUND**:

- Were you reluctant towards the ultrasound? YES/ NO

If yes, why? …..............................................................................................................................

- Shortly describe your experience with/ opinion on the ultrasound?

.....................................................................................................................................................

.....................................................................................................................................................

.....................................................................................................................................................

.....................................................................................................................................................

**MAMMOGRAPHY:**

- Were you reluctant towards the mammogram? YES/ NO

If yes, why? ............................................................................................................................

- Despite having the complaint in one breast, there was also a mammogram performed on the other breast. What did you think about this (see options below)?
  - Reassuring
  - Unnecessary, painful examination
  - Superfluous X-radiation
  - Neutral
- Shortly describe your experience with/ opinion on the mammogram?

.....................................................................................................................................................

.....................................................................................................................................................

.....................................................................................................................................................

.....................................................................................................................................................

Room for suggestions:

............................................................................................................................................................................................................................................................................................................................................................................................................................................................................................................................................................................................................................................................................

Thank you very much for completing this survey!
