# Supplementary material for: Patients’ perceptions of targeted breast ultrasound and digital breast tomosynthesis in the diagnostic setting: A mixed methods study
Source: PLoS One. 2024 Aug 14;19(8):e0308840. doi: 10.1371/journal.pone.0308840 (PMC11324127; doi:10.1371/journal.pone.0308840)
Supplement: S2 Appendix — (DOCX) [file pone.0308840.s002.docx]

**Interview prompt questions**

When you visited the Radiology department, the order of imaging examinations was reversed, first performing ultrasound followed by mammography. What was your experience with the diagnostic imaging procedure?

- What did you think was pleasant? Why?
- What did you think was unpleasant? Why?
